# The combination of glucagon-like peptide-1 receptor agonists and sodium–glucose cotransporter-2 inhibitors: a narrative review of existing meta-analysis

**DOI:** 10.1007/s00592-026-02666-9

**Published:** 2026-02-18

**Authors:** Angelo Avogaro, Matteo Neccia, Marina Delfini, Aldo Franculli, Giulia Enea, Anna Magli, Joshua Bemporad, Gian Paolo Fadini

**Affiliations:** 1 Department of Medicine, University of Padova, Padua, Italy; 2 Azienda Ospedaliero-Universitaria Policlinico Umberto I, “Sapienza” University of Rome, Rome, Italy; 3 Cardiology Unit, S. Hospital, Rome, Italy; 4 Nephrology Unit, Ospedale Dei Castelli, Rome, Italy; 5 Cardiology Unit, Ospedale Santa Croce, Fano, Italy; 6 Nephrology Unit, Ospedali Runiti, Ancona, Italy; 7 Endocrinology and Metabolism Unit Università Campus Bio-Medico,i,, Rome, Italy

**Keywords:** GLP-1 receptor agonists, SGLT2 inhibitors, Combination therapy, Cardiovascular outcomes, Diabetic kidney disease

## Abstract

Glucagon-like peptide-1 receptor agonists (GLP-1RAs) and sodium-glucose cotransporter-2 inhibitors (SGLT2i) have profoundly reshaped the therapeutic landscape of type 2 diabetes mellitus (T2DM). In this narrative review, we critically appraise the published literature on the combined effects of these two classes on cardiovascular outcomes, heart failure, and kidney disease. Randomized controlled trials indicates that combination therapy achieves greater reductions in HbA1c compared with either agent alone. Meta-analyses demonstrate that both GLP-1RAs and SGLT2i reduce the risk of major adverse cardiovascular events (MACE) to a comparable extent, while SGLT2i provide superior benefits in mitigating hospitalization for heart failure and slowing chronic kidney disease progression. However, cardiovascular outcome trials (CVOTs) have not demonstrated a synergistic or additive protective effect of combined therapy on MACE or renal endpoints. In contrast, real-world evidence suggests incremental benefits across MACE, heart failure, and renal outcomes, supporting the hypothesis that complementary mechanisms of action may translate into broader protection in clinical practice. In this context, the aim of the present work is to summarize the existing meta-analyses that have evaluated both real-world studies and randomized controlled trials assessing the combination therapy of GLP-1 receptor agonists and SGLT2 inhibitors.Globally, GLP-1RAs and SGLT2i should be regarded as complementary rather than alternative therapeutic strategies. Randomized evidence supports the individual efficacy of each class and real-world data increasingly endorse their combined use to optimize cardiovascular and renal protection in patients with T2DM.

## Introduction

Glucagon-like peptide-1 receptor agonists (GLP-1RAs) and sodium-glucose cotransporter-2 inhibitors (SGLT2i) have become central to the management of type 2 diabetes mellitus (T2DM). Beyond glycaemic control, both drug classes offer significant cardiovascular and renal benefits, as demonstrated in numerous clinical trials, underscoring their role in comprehensive diabetes care[1].

GLP-1RAs have been shown to significantly reduce major adverse cardiovascular events (MACE) in individuals with T2DM, and even those with overweight or obesity without diabetes [2]. The recent STRIDE trial evaluated semaglutide's impact on functional outcomes, revealing improved walking distance in patients with T2DM and symptomatic peripheral artery disease [3]. Importantly, cardiovascular benefits of GLP-1RAs appear to be independent of glycaemic control, with evidence supporting MACE risk reduction in non-diabetic populations [4]. In addition to cardiovascular benefits, post-hoc analyses of trial data suggested that GLP-1RAs may confer renal protection, as clinical trials have consistently reported reductions in composite renal outcomes [5].

Similarly, SGLT2 inhibitors have demonstrated robust cardiovascular and renal benefits, including reductions in cardiovascular events and all-cause mortality, with consistent effects across the drug class and irrespective of diabetes status [6, 7] Their metabolic benefits—such as weight loss, improved lipid profiles, and reduced visceral adiposity—contribute to a favourable cardiovascular risk profile, countering the pro-atherogenic state typical of T2DM [8]. They also reduce the risk of hospitalization for heart failure (hHF) [9], and the risk for the loss of renal function [10, 11].

As a result, current clinical guidelines for T2DM, kidney disease, and cardiovascular care strongly recommend the use of GLP-1RAs and SGLT2is in patients with T2DM who have, or are at high risk for, atherosclerotic cardiovascular disease (ASCVD), heart failure (HF), or chronic kidney disease (CKD).

Objectives of this narrative review are to summarize major meta-analysis findings on combination therapies, including real-world evidence (RWE) and cardiovascular outcome trials (CVOTs), with a focus on metabolic, cardiovascular, and renal endpoints.

## Effects of combination therapy on intermediate endpoints and side effects according to meta-analyses of randomized trials

We analysed data from 6 meta-analyses (Table 1), which assessed the effect of the combination therapy on HbA1c and body weight. The combination exerts robust effects on glycaemic control, although the magnitude of benefit varies depending on the comparator. Across all analyses, the addition of a GLP-1RA to ongoing SGLT2i therapy consistently produced a clinically meaningful reduction in HbA1c. Castellana et al. found that combination therapy was associated with a greater reduction in HbA1c of -0.74% compared to SGLT2i alone, while Chen Li et al. reported a reduction of 0.77% compared to monotherapy [12, 13]. Guo et al. noted a 1.32% reduction versus control/placebo, while Tuersun et al. confirmed a significant decrease (SMD -0.45%) versus SGLT2i monotherapy [14, 15]. Conversely, adding an SGLT2i to a GLP-1RA did not yield a statistically significant further reduction in HbA1c (SMD –0.11%), suggesting that the glycaemic benefit of combination therapy is predominantly driven by the GLP-1RA component. Notably, Chen Li et al. reported that this effect diminished over time, becoming less pronounced after one year of treatment.

**Table 1:** Summary Table of All Meta-Analyses

Meta-analysis	N. of studies	Total subjects
	Metabolic end-points	
Patoulias et al	3 RCTs	1042
Castellana et al. (2019)	4 RCTs	1610
Guo et al. (2020)	11 studies	1604
Tuersun et al. (2024)	7 RCTs	Not reported
Chen Li et al. (2022)	8 RCTs	1895
Yaping et al. (2024)	15 studies	11,679
	Observational study	
Aftab Ahmad et al. (2024)	8 studies (meta‑analysis subset)	Not stated
Collombin et al. (2025)	15 cohort studies	991,731
	CVOT studies	
Apperloo et al. (SMART-C, 2024)	12 RCTs: DAPA-CKD, EMPA-KIDNEY, DAPA-HF, EMPEROR-Reduced, EMPEROR-Preserved, DELIVER + 6 ASCVD trials	73,238
Neuen et al. (Circulation 2024)	3 RCTs: FLOW, AMPLITUDE-O, Harmony Outcomes	17,072

Beyond glycaemic control, a key advantage of this combination lies in its multifaceted metabolic benefits. Several studies have shown substantial weight reduction with combined therapy. Castellana et al. demonstrated -1.61 kg greater loss vs. SGLT2i alone, while Guo et al. − 0.93 kg reduction vs. controls [12, 14]. Chen Li et al. reported a significant reduction (SMD − 0.36 kg), though this effect also weakened after one year [13]. The meta-analysis by Tuersun found no significant difference in body weight reduction when comparing the combination therapy to either GLP-1RA monotherapy or SGLT2i monotherapy: this finding challenges the assumption of a synergistic or additive effect on weight loss [15].

Combination therapy also tends to improve lipid profiles. Multiple analyses have demonstrated significant reductions in LDL cholesterol, while Yaping et al. reported both lower LDL and higher HDL cholesterol in elderly patients [16]. It should be noted that the extent of weight loss achieved with GLP-1RA add-on to SGLT2i appears anyway modest compared to the efficacy of newer treatments for weight management, including dual incretin receptor agonists.

Regarding safety, most meta-analyses concluded that combination therapy does not increase the risk of hypoglycaemia compared with monotherapy. However, Guo et al. and Chen Li et al. reported a modestly higher risk, with relative risks (RR) of 2.22 and 1.82, respectively [13, 14]. An increased risk of GI side effects, including nausea, vomiting, and diarrhoea, is consistently reported: these events are attributed to the GLP-1RA component of the therapy, while an elevated risk of genital infections is associated with the SGLT2i component.

In summary, combination therapy produced consistent and clinically meaningful reductions in HbA1c, averaging about 0.7–1.3% compared with monotherapy or placebo. Most analyses indicate that the glycaemic effect is mainly driven by the GLP-1RA component and may attenuate over time. There are several reasons to explain this limited beneficial effect on HbA1c (and also on body weight). The first is intrinsic to the complexity of pathophysiology of Type 2 diabetes; second, most of the combination studies have been performed using exenatide, one of the least effective GLP-1RA in terms of HbA1c and body weight reduction; third, combination therapy may work differently in different phenotypes of type 2 diabetes[17].

Combination therapy also modestly improves weight, blood pressure, and lipid parameters, with reductions in body weight of approximately 1–1.5 kg versus SGLT2i alone, though evidence for additive effects on weight loss is inconsistent. Lipid profiles improve, particularly through lower LDL and higher HDL levels. Among adverse events, hypoglycaemia risk is generally comparable to monotherapy[18]. This is reassuring also in the light that neither classes of drugs induce hypoglycemia. However, higher incidence of hypoglycemia can be a possibility in the presence of insulin-coadministration. There appears to be no interaction between GLP-1RA and SGLT2i in modifying their safety profile, with gastrointestinal adverse events attributable to GLP-1RAs and genital infections to SGLT2i.

## Effects of combination therapy on hard endpoints according to meta-analyses of observational studies

In the context of real-world observational data, we have specifically analyzed 2 meta-analysis: Ahmad & Sabbour assessed 13 studies, while Colombijn et al. included 18 cohort studies [19, 20]. It is important to note that only two studies were overlapping the two meta-analysis: the reason for this remains unclear.

Colombijn et al. found that combination therapy may lead to a relative risk reduction by 44% in MACE %, by 35% for stroke, and by 38% for myocardial infarction. For kidney function, Ahmad & Sabbour reported a significant reduction in albuminuria only in one study, while Colombijn et al. reported a lower risk of kidney composite endpoint (RR 0.48].

Both metanalyses found no evidence of worsening safety risks with combination therapy, although data were limited. Ahmad & Sabbour note that most commonly reported adverse events were genital/urinary tract infections, gastrointestinal issues, and polyuria. Major adverse effects like ketoacidosis or pancreatitis were not reported. Specific data from some events of particular interes were reported by Colombijn et al., including severe hypoglycaemia (4% in the combination group vs. 3% in the monotherapy group) or diabetic ketoacidosis (< 1% in both combination and monotherapy groups).

These studies acknowledge significant limitations inherent to their reliance on observational and real-world data, which impacts the degree of the evidence. Sixteen of the 18 cohort studies in the Colombijn et al. review was judged to have "severe or critical methodological limitations". Despite the acknowledged limitations, these two metanalyses reach a consistent conclusion: the combination of SGLT2i and GLP-1RA is associated with significantly lower all-cause mortality and superior cardiorenal outcomes compared to monotherapy in patients with T2DM.

The findings from these real-world data form supportive evidence for current clinical guidelines that recommend combining these agents for patients with T2DM and high cardiorenal risk.

## Effects of combination therapy on hard endpoints based on meta-analyses of CVOTs

Large-scale collaborative meta-analyses of CVOTs have examined whether the benefits of one drug class are maintained when the other is used concurrently. We have identified 2 meta-analyses assessing the combination therapy in CVOTs (Table 1). Apperloo et al. conducted a collaborative meta-analysis of randomised, double-blind, placebo-controlled, event-driven outcome trials within the SGLT2 Inhibitor Meta-Analysis Cardio-Renal Trialists’ Consortium (SMART-C Meta-Analysis, 2024)[21]. They included 12 SGLT2i trials with 73,238 participants, of whom 3,065 were on a GLP-1 RA: they found that the benefits of SGLT2i on MACE were consistent regardless of background GLP-1 RA use (HR = 0.81 in patients receiving GLP-1 RA vs HR = 0.90 in those not receiving GLP-1RA) with no significant heterogeneity (p = 0.31). Effects were highly consistent for the composite outcome of hHF or CV death (HR 0.76 vs. 0.78; p-heterogeneity = 0.90). Risk reduction for CKD progression was nearly identical between the two groups with/without background GLP-1RA (HR 0.65 vs. 0.67; p for heterogeneity = 0.81). It must be acknowledged that despite similar point estimate of the HRs and the absence of statistically significant heterogeneity, SGLT2i in combination with GLP1Ra displayed HR with confidence intervals below 1.0 for chronic kidney disease progression, but not for hospitalization for heart failure or cardiovascular death.

Neuen et al. included in the analysis 3 GLP-1RA trials with 17,072 participants, of whom 1,743 were on an SGLT2i. They found consistent benefits for GLP-1RA regardless of background SGLT2i use [22]. Their meta-analysis showed that a reduced risk for MACE with GLP-1RA was consistent in patients receiving SGLT2i (HR = 0.77) and those not (HR = 0.79; p-heterogeneity = 0.78). The results were consistent also for hHF (HR 0.58 vs. 0 = 73; p-heterogeneity = 0.26), while the risk for composite kidney outcome did not vary by SGLT2i use.

These findings provide powerful evidence that the two drug classes operate through independent pathways, making their benefits additive when used in combination.

## Final outlook and conclusions

**Fig. 1:**
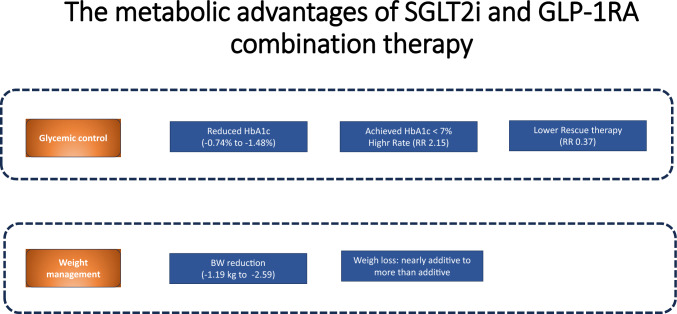
The summarized effects of GLP_1RA and SGLT2i combination on HbA1c (left) and body weight (right) in different meta-analysis

**Fig. 2:**
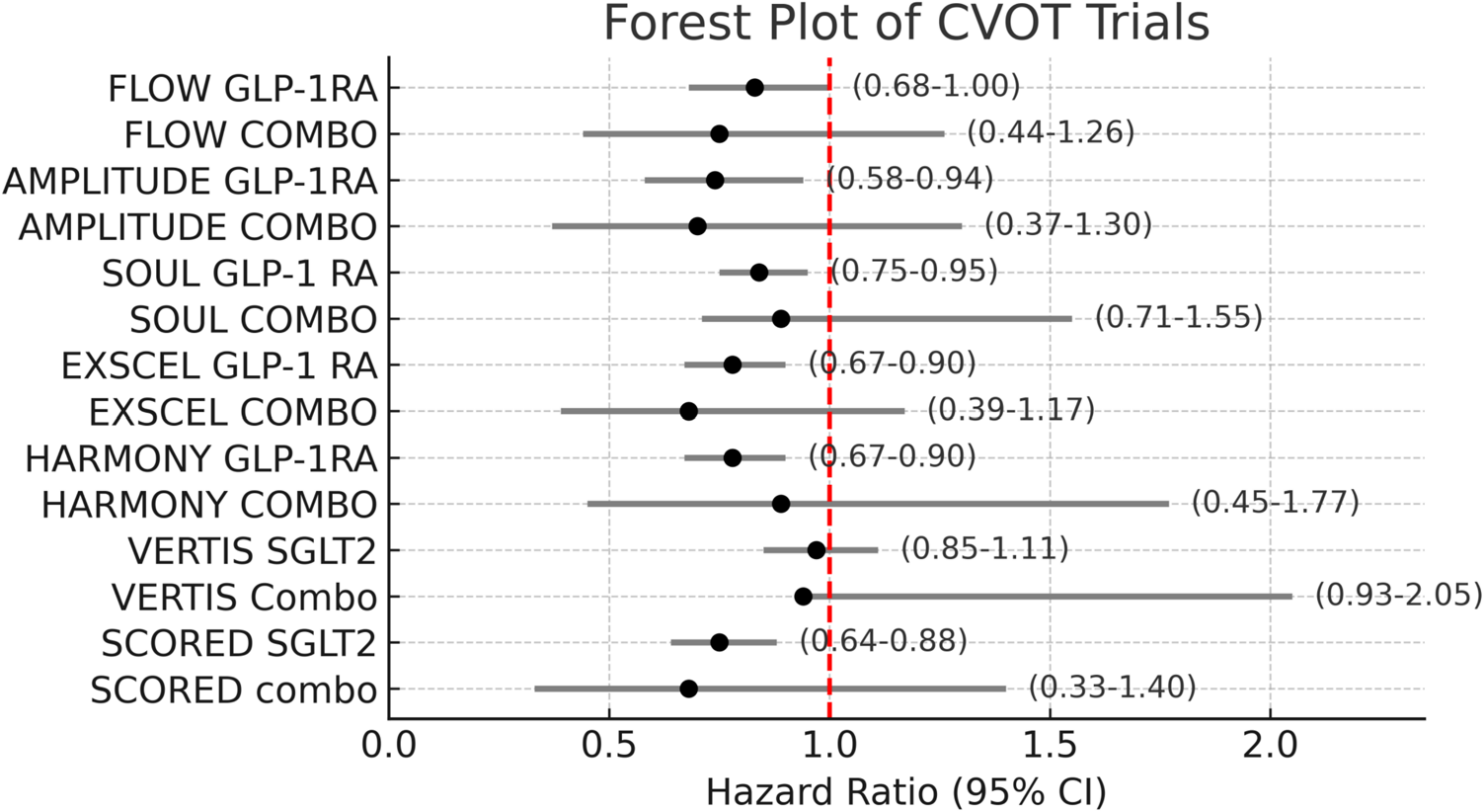
The reported HR (with 95% CI) for MACE the RWE in the different trials for single and combination therapies

SGLT2i and GLP-1RA exert cardio-renal protection through distinct, primarily glucose-independent pathways, making their combined use a logical strategy to improve cardiometabolic and renal outcomes. Their complementary mechanisms suggest that their combination has the potential for greater cardiovascular and renal protection than either agent alone Figure 1. Although the results from observational studies are consistent, the degree of evidence is inherently limited by the non-randomized design, while evidence from CVOTs is far from being conclusive. Figure 2 illustrates that CVOTs consistently demonstrate a reduction in major adverse cardiovascular events (MACE) with either GLP-1RA or SGLT2i monotherapy, whereas combination therapy shows a uniform but non-statistically significant reduction. This pattern is observed in trials where patients were randomized to either GLP-1RA or SGLT2i treatment. No randomized trial has evaluated the effects on hard outcomes of simultaneous initiation of GLP-1RA and SGLT2i versus an alternative treatment approach.

**Fig. 3:**
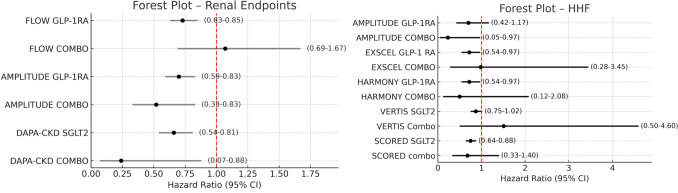
The reported HR (with 95% CI) for renal end-points (left) and hospitalization for hHF (right) in CVOT

For renal outcomes (Fig. 3), comparisons are limited due to differences in endpoint definitions across studies: nevertheless, CVOTs results suggest substantial kidney protection with both monotherapy and combination therapy, except for the FLOW trial. This trend, however, is not seen for hHF (Fig. 3). These results are largely expected since both SGLT2i and GLP-1RA positively influence renal outcome with different mechanisms, whereas GLP-1RA exert minor effects on hHF,, except in non-diabetic patients with obesity [23].

Several factors may explain why CVOTs have not demonstrated more robust or statistically significant results with combination therapy. One possibility pertains their combined effect on HbA1c and body weight. As an example, in the DURATION-8 study, while combination therapy produced greater HbA1c and body weight reductions, the effect was less than additive compared to the sum of individual drug effects, most likely because the efficacy of glucose-lowering therapies can depend on baseline glycaemic control [24]. Specifically in this trial, and in no other trials, SGLT2i treatment was combined with exenatide treatment. The combination therapy produced substantially greater weight loss than either agent alone. In the DURATION-8 trial, patients treated with exenatide ER plus dapagliflozin achieved nearly double the weight reduction observed with each monotherapy, with numerically larger effects in individuals with higher baseline BMI. Nonetheless, the overall impact on body weight remained less than strictly additive. It is known that exenatide, an exendin‑based molecule, provides smaller average weight effects than semaglutide in head‑to‑head and pooled comparisons—likely reflecting differences in receptor potency, exposure profile, and possibly central nervous system engagement [25].

Findings on combination therapy in CVOTs must be interpreted cautiously. Differences in study design, together with the relatively small number of participants receiving combined treatment, may limit the ability to detect synergistic effects. Importantly, in most CVOTs the combined use of the two drug classes was not a prespecified endpoint. For instance, in the FLOW trial, only 277 participants in the combination group compared to 1,490 receiving semaglutide alone. Assuming a 10% incidence of the primary endpoint and a 15% relative risk reduction, the statistical power to detect a significant difference with just 277 patients would be only 11%. Therefore, a RCT designed specifically to compare combination therapy with monotherapy is essential to draw definitive conclusions. Until such data are available, the evidence relies on post-hoc analyses, which are limited by small sample sizes and statistical inflation of type I error.

In conclusion, available data suggest that GLP-1RAs and SGLT2i should not be viewed as rivals, but rather as complementary therapies offering synergistic cardiovascular and renal benefits in the management of T2DM. Their distinct mechanism of action and non-overlapping effects suggest potential for additive benefits when used together. This is more convincingly reported in the RWE studies but not in the post-hoc analysis of the CVOTs, which are characterized by a small number of patients receiving the combination treatment.

Finally, combination therapy is more feasible in high-income settings, where healthcare systems can sustain the associated costs. As always, treatment decisions should be individualized, considering kidney function, comorbidities, adverse-event profile, costs and reimbursement, and patient preferences.
